# PTEN inhibitor bpV(HOpic) confers protection against ionizing radiation

**DOI:** 10.1038/s41598-020-80754-8

**Published:** 2021-01-18

**Authors:** Ankit Chauhan, Dhananjay Kumar Sah, Neeraj Kumari, Namita Kalra, Ravi Soni, Anant Narayan Bhatt

**Affiliations:** Institute of Nuclear Medicine and Allied Sciences, Brig. S. K. Mazumdar Road, Timarpur, Delhi, 110 054 India

**Keywords:** Cell signalling, Cell death, Apoptosis, DNA damage and repair

## Abstract

Exposure to Ionizing radiation (IR) poses a severe threat to human health. Therefore, there is an urgent need to develop potent and safe radioprotective agents for radio-nuclear emergencies. Phosphatidylinositol-3-kinase (PI3K) mediates its cytoprotective signaling against IR by phosphorylating membrane phospholipids to phosphatidylinositol 3,4,5 triphosphate, PIP3, that serve as a docking site for AKT. Phosphatase and Tensin Homolog on chromosome 10 (PTEN) antagonizes PI3K activity by dephosphorylating PIP3, thus suppressing PI3K/AKT signaling that could prevent IR induced cytotoxicity. The current study was undertaken to investigate the radioprotective potential of PTEN inhibitor (PTENi), bpV(HOpic). The cell cytotoxicity, proliferation index, and clonogenic survival assays were performed for assessing the radioprotective potential of bpV(HOpic). A safe dose of bpV(HOpic) was shown to be radioprotective in three radiosensitive tissue origin cells. Further, bpV(HOpic) significantly reduced the IR-induced apoptosis and associated pro-death signaling. A faster and better DNA repair kinetics was also observed in bpV(HOpic) pretreated cells exposed to IR. Additionally, bpV(HOpic) decreased the IR-induced oxidative stress and significantly enhanced the antioxidant defense mechanism in cells. The radioprotective effect of bpV(HOpic) was found to be AKT dependant and primarily regulated by the enhanced glycolysis and associated signaling. Furthermore, this in-vitro observation was verified in-vivo, where administration of bpV(HOpic) in C57BL/6 mice resulted in AKT activation and conferred survival advantage against IR-induced mortality. These results imply that bpV(HOpic) ameliorates IR-induced oxidative stress and cell death by inducing AKT signaling mediated antioxidant defense system and DNA repair pathways, thus strengthening its potential to be used as a radiation countermeasure.

## Introduction

The exposure of the whole body to the high doses of IR during the events of nuclear and radiation accidents poses a serious threat to human health. The deleterious effects of IR are orchestrated through a series of cellular responses leading to direct damage and oxidation of the biological molecules^1^. The severity of the damage incurred due to radiation injury depends upon the type of tissue and the radiation dose to which the individual was exposed. The hematopoietic system and villous epithelium of the small intestine, due to their high proliferation rate, are the most vulnerable tissues to the damaging effect of radiation^2^. Amifostine, well documented for its cytoprotective effect against radio- and chemo-therapy induced toxicity, has been approved by US FDA for the management of radio- and chemo- therapy related normal tissue cytotoxicity^3^. Apart from Amifostine, some therapeutic drugs like Neupogen/Neulasta are recommended for low dose exposure in USA^4, 5^. However, dose-related adverse effects and unfavorable administration route limited the development of Amifostine as a radiation countermeasure drug^6^. There is no approved radioprotective drug available over the counter currently, suitable for field application; therefore, there is an urgent need to develop radioprotective drugs by utilizing established and potential cellular targets, to contend the detrimental effects of IR.

Exposure to IR leads to the activation of membrane-bound receptor tyrosine kinases (RTKs) (e.g., erbB family of receptors like EGFR). These activated receptors, in turn, enhances the activities of the RAS family of transducer molecules that further activate multiple signal transduction pathways such as the PI3K/AKT pathway, Ras-mitogen activated protein kinase (MAPK)/extracellular signal-related (ERK1/2) kinase pathway, the signal transducer and activator of transcription (STAT) pathway, and the Nuclear Factor kappa-light-chain-enhancer of activated B cells (NFκB) pathway^7–9^. IR induces phosphorylation of certain tyrosine residues in the cytoplasmic domain of RTKs, which further phosphorylates PI3K and activates downstream PI3K–AKT signal transduction pathway^10^. IR induced PI3K–AKT signaling has been investigated in detail and known to confer radioresistance^11–15^. The role of activated PI3K–AKT signal transduction pathway in regulating various cellular functions such as cell proliferation, growth, metabolism, DNA double-strand break (DSB) repair, and survival are well established^16–18^. Phosphatidylinositol 3-kinase family members regulate a variety of cellular functions by phosphorylating membrane phospholipids (phosphatidylinositol 4,5 bisphosphate, PIP2 to phosphatidylinositol 3,4,5 triphosphate, PIP3) in response to extracellular cues, which serve as a docking site for the proteins with pleckstrin homology (PH) domain such as AKT and PDK1. Upon docking at PIP3, AKT kinase is activated by phosphorylation at Thr 308 and Ser 473 residues by PDK1 and mTORC1 kinases, respectively. The activated AKT phosphorylates a plethora of downstream substrates that regulate cell survival, proliferation, and growth^19^.

Phosphatase and Tensin Homolog gene (PTEN) encodes a PIP3 3′-phosphatase that counters the activity of PI3K–AKT signaling pathway by dephosphorylating PIP3 to PIP2 thereby meticulously regulating a myriad of cellular processes like proliferation, growth, metabolism, and survival^20, 21^. The role of PTEN in regulating cellular oxidative stress has been elucidated, and pharmacological inhibition of PTEN shown to provide a therapeutic gain in several experimental models where oxidative stress plays a significant role. PTEN inhibition was shown to be protective against hepatic and cerebral ischemia/reperfusion (I/R) injuries^22, 23^. In an oleic acid-induced model of acute lung injury, PTEN inhibition was shown to protect lung parenchyma^24^. PTEN inhibition is also shown to reduce the myocardial infarct size and alleviate the I/R injury^25^.

Multiple studies have shown the human use of vanadium and vanadium-based compounds to be insulin-mimetic as well as cardioprotective in diabetes, the exact mechanism of action remains elusive^26, 27^. One of the widely accepted mechanisms of the observed insulin mimesis in vanadium is the inhibition of protein tyrosine phosphatases like PTEN and activation of the insulin receptor as well as tyrosine kinases^28^. Based on these findings, several peroxovanadium compounds have been synthesized that are potent PTEN inhibitors^29, 30^. Interestingly, few vanadium-derivative PTEN inhibitors are also shown to activate primary follicles in-vitro and in-vivo^31, 32^ and are currently under clinical trials in patients with primary ovarian insufficiency to improve the success rate of in-vitro fertilization (ClinicalTrials.gov Identifier: NCT04131244 and NCT02322060). Despite this, currently, there is no report exists that investigated the use of PTENi against the harmful effects of ionizing radiation. Given the cytoprotective effect of PTENi against several oxidative injuries and the role of PTEN in regulating the radiation response of cells, PTEN targeting is being proposed here as a radiation countermeasure.

In this study, we investigated the protective effects of bisperoxovanadium inhibitor [bpV(HOpic)] of PTEN against IR. We observed that the PTEN inhibitor protected mouse cell lines of different tissue origins against the IR induced cell death. Further, the PTEN inhibitor augmented energy metabolism and enhanced DNA repair processes. The administration of PTEN inhibitor in mice before the lethal exposure to IR protects them against IR-induced mortality.

## Materials and methods

### Cell culture

The mouse lung fibroblasts (NIH-3T3), human embryonic mouse normal monocyte macrophages (Raw 264.7), and human embryonic intestinal cells (INT-407) were obtained from NCCS, Pune, India and cultured in their respective growth medium (mainly HGDMEM) supplemented with 10% fetal bovine serum. All experiments were carried in exponentially growing cell cultures.

### Treatment

All the experiments were performed in 24 well plates, 60 mm, and 35 mm tissue culture dishes. Cells in their Log phase were treated with either γ-radiation or PTENi following overnight incubation at 37 °C in 5% CO_2_ in the incubator. For PTENi treatment, cells were pretreated for 1 h before irradiation with either 100 μmol/L or 500 μmol/L of bpV(HOpic) from Sigma-Aldrich. The dose–response analysis (1 Gy, 2 Gy, 4 Gy, 6 Gy, and 8 Gy) of γ-radiation was carried out in NIH-3T3, Raw 264.7, and INT-407 cell lines. Further, all experiments were carried out at a single radiation dose of 2 Gy (Raw 264.7), 4 Gy (NIH-3T3), 6 Gy (INT-407). All in-vitro radiation treatments were given using institutional Telecobalt Facility (Bhabhatron II, Panacea Medical Technologies Pvt. Ltd, India) with ^60^Co as a source, 35 × 35 cm field, 80 cm SSD; and a dose rate ranging from 1.0 to 0.676 Gy/min.

### Cell viability

For cell viability assays, cells were seeded 24 h before PTENi treatment into 96-well plate at a density of 2500–3000 cells per well in 200 μl growth medium. Cells were treated with PTENi (10 nmol/L–10 μmol/L) one hour before irradiation. Cell viability was measured using sulphorhodamine-B stain (SRB) (Sigma-Aldrich). At 48 h after irradiation, the growth medium was removed, and cells were fixed in 10% (w/v) trichloroacetic acid for 1 h at 4 °C. Cells were washed twice with double deionized water to remove excess fixative, air-dried and incubated in SRB stain solution [0.4% (w/v) SRB prepared in 1% (v/v) acetic acid] at 37 °C for 30 min. The excess stain was washed with 1% acetic acid, and protein-bound dye was dissolved in 10 mM pH 10 Tris base solution. The optical density was read at 564 nm on an automated microplate spectrophotometer.

### Cell proliferation

Approximately 1 × 10^5^ cells were seeded in a PD 35 mm for cell proliferation studies. The cells were allowed to grow in growth medium post-treatment, harvested by trypsinization, and counted on hemocytometer at indicative timepoints. The cell proliferation was calculated by assessing the increase in cell number and cell proliferation index, calculated as P = N_t_/N_0,_ where N_t_ denotes cell number at time t, and N_0_ is cell number at the time of treatment.

### Macrocolony assay

Clonogenic survival of cells pretreated with PTENi before irradiation was analyzed by macrocolony assay, as described earlier^33^. Briefly, varying number of exponentially growing cells (100–3200, depending on the plating efficiency of different cell lines and irradiation dose) were seeded in triplicates in 60 mm Petri dishes 24 h before treatment. Cells were treated with PTENi one hour before irradiation and were allowed to form colonies at 37 °C in the CO_2_ incubator. After 7–10 days, when macrocolonies were visible, cultures were terminated, fixed in 10% Methanol, and stained with 1% crystal violet. Stained colonies of at least 50 cells were scored, and plating efficiency PE was calculated as PE = [No. of colonies counted/No. of cells plated]/100. The surviving fraction SF was calculated as SF = PE of Treated group/PE of control.

### Cell death assays

Acridine Orange and Ethidium bromide (EtBr) staining was performed in NIH-3T3 cells to account for the apoptotic cell, as described previously^34^. Briefly, cells were cultured in 96 well plates overnight prior to treatments. At indicative timepoints, plates were centrifuged briefly and incubated in 1:1 Acridine Orange and EtBr (Sigma-Aldrich) to a final concentration of 10 μg/ml each and incubated at room temperature for 10 min. Fluorescence was visualized under an Olympus fluorescence microscope, and percent of apoptotic cells out of total cells per field were calculated. Caspase-3 and -7 activity was studied using Cell Event Caspase 3/7 activity probe (Invitrogen, Ref. C10423) following manufacturer’s protocol and percent positive cells out of total cell per field represented as Caspase-3 and -7 positive. For flow cytometry-based cell death analysis, we performed AnnexinV-PI (Invitrogen) staining following the manufacturer’s instructions and cells were analyzed using BD FACS ARIA.

### γ-H_2_AX foci formation assay

The residual DNA-double stranded breaks were determined by the γ-H_2_AX foci formation assay. Briefly, for γ-H_2_AX immunostaining, 0.1 × 10^5^ cells were cultured on coverslips, at indicative timepoints cells were washed with ice-cold PBS, and permeabilized with 0.1%v/v Triton X-100 for 10 min at room temperature and washed again with ice-cold PBS. Non-specific protein binding was blocked with 4% BSA for 60 min at room temperature. Primary antibody (γ-H_2_AX Cell Signaling Technologies, 1:800) incubation was done for 1 h at room temperature followed by washing and secondary incubation with FITC conjugated secondary antibody (Sigma-Aldrich) for 45 min at room temperature. Cells were rewashed and mounted on a clean slide with antifade mount solution with DAPI (Invitrogen, Cat #P36931). Immunofluorescence was acquired using MetaCyte γ-H_2_AX foci scan software of an automated Metafer microscope (Metasystem, Germany). Roughly 150–200 images were analyzed with and Metafer (Version) and verified by manual counts.

### 53BP-1 foci formation assay

53BP-1 foci formation was carried out in HEK cells stably transfected with a 53BP1-GFP plasmid (a gift from Dr. Deepak Saini’s Laboratory, Indian Institute of Sciences, Bangaluru, India) using Lipofectamine 2000 (Invitrogen). For imaging, roughly 0.075 × 10^6^ cells were cultured on sterile coverslips in a 35 mm PD with 2 ml growth medium, 24 h before treatment. 48 h post-treatment GFP fluorescence was observed, and images were captured using a fluorescence microscope (Olympus IX51, Japan) with 20X (objective) × 10X (eyepiece) magnification. Average foci formed per cell were counted.

### Micronuclei assay

Approximately 1 × 10^5^ cells were grown in a PD 35 mm for micronuclei assay, as described earlier^33^. Cells were washed and fixed at indicated timepoints in Carnoy’s fixative (3:1 v/v, Methanol: Acetic Acid) at 4 °C overnight. Fixed cells were dropped on pre-chilled glass slides, air-dried, and stained with 10 μg/ml of DNA specific fluorochrome, diamidino-2-phenylindole dihydrochloride, DAPI, prepared in phosphate buffer (0.05 M Na_2_ HPO_4_·2H_2_O, 0.05%, Tween-20, 0.01 M citric acid, pH 7.4) in dark, at room temperature for 15 min. Stained slides were rinsed in PBS, mounted in an aqueous mounting medium (PBS-Glycerol, 1:1 v/v). Micronuclei fraction was scored using the criteria suggested by Countryman and Heddle^35^, from ~ 1000 cells per group from triplicate slides under a fluorescence microscope using a UV excitation filter. Micronuclei fraction (MF), i.e., the percentage of cells with micronuclei, was then calculated as MF(%) = N_m_/N_t_ × 100, where N_m_ is the number of cells with micronuclei, N_t_ is the total number of cells analyzed.

### Measurement of reactive oxygen species

Intracellular reactive oxygen species (ROS) were assessed using the oxidant-sensitive fluorescent probes; CM-H_2_DCFDA for total ROS and MitoSOx Red for mitochondrial ROS (Molecular probes), following the manufacturer’s protocol. Briefly, at indicative timepoints, cells were washed with ice-cold PBS, trypsinized and incubated in probe buffer (1 mM CaCl_2,_ 1 mM MgCl_2,_ 5 mM Glucose) with either probe (20 μM CM-H_2_DCFDA or 5 μM MitoSox Red) at 37 °C for 30 min. Cells were rewashed to remove the excess probe and resuspended in probe buffer. Fluorescence was read on a BD FACS ARIA flow cytometer.

### Glucose uptake

Glucose uptake was measured using fluorescently-labeled deoxyglucose analog, 2-NBDG (Molecular Probes). At indicative timepoints, cells were washed, incubated with 100 μM 2-NBDG at 37 °C for 30 min, and washed again to remove the excess probe. Cells were scraped and resuspended in cold PBS, and fluorescence was read with a BD FACS ARIA flow cytometer.

### Protein carbonylation

Total protein carbonyl content was estimated as described earlier^36, 37^ with some minor modifications. Briefly, cells were cultured in 75 cm tissue culture flasks 24 h before treatment. Cells were harvested on the ice at indicative time points post-treatment, lysed, and total protein was quantified using the BCA method. An equal amount of protein (1 mg/sample) was precipitated using 20% TCA followed and treated with 10 mM DNPH at room temperature for 60 min. The DNPH protein derivative was again precipitated with 20% TCA and washed twice with ethanol: ethyl acetate (1:1) and finally dissolved in 6 M Guanidium Hydrochloride. Absorbance was read at 370 nm on a 96-well spectrophotometer, and total carbonyl content was estimated and represented as:

1. Protein carbonyl nmol/ml = [Absorb/Ex.Co.(0.022)] * GdmHCL Vol./Vol. of protein.
2. Carbonyl content (nmol/mg) = (Protein carb. nmol/ml)/Protein mg/ml.

### Lipid peroxidation assay

Lipid peroxidation levels were assayed by measuring the levels of Thiobarbituric acid reactive substances (TBARS), malondialdehyde (MDA) in cells at indicative timepoints as described previously^6^. Briefly, cells were harvested at indicative timepoints and homogenized in ice-cold Tris-KCl buffer ((10 mM Tris-HCl, 150 mM KCl, pH 7.4). One volume of clear homogenate was heated for 45 min in a water bath with two volumes of 0.37% w/v Thiobarbaturic Acid and 15% w/v Trichloroacetic acid. After that, the absorbance of clear supernatant was read at 532 nM on a 96 well-plate spectrophotometer. Using a molar absorption coefficient of 155 mM^−1^ cm^−1^, lipid peroxidation levels were calculated as nanomoles of MDA formed per milligram of protein.

### siRNA transfection

NIH-3T3 cells were transfected with scrambled or AKT1/2 siRNA (Santacruz Biotechnology, Inc, USA) for the knockdown of gene expression. siRNA transfection was performed using Lipofectamine 2000 (Invitrogen) as per manufacturer’s protocol. Briefly, 0.1 × 10^6^ cells were seeded in a 6-well plate and allowed to grow for 24 h. Transfection was performed in Opti-MEM (serum and antibiotic-free medium) for 4-h followed by a 24-h recovery in medium supplemented with serum.

### Western blot analysis

Cells were lysed in RIPA lysis buffer [45 mM HEPES, 50 mM KCL, 5 mM EDTA, 50 mM NaF, 1 mM Na_3_VO_4_, 0.1% Triton-X100, 1 mM PMSF, 1 mM Bezamidine, 1 × Protease (Pierce Biotechnology) and phosphatase (Roche) inhibitors cocktail] and total protein concentration in the lysate was quantified using BCA-Assay kit (Pierce Cat #23227). Equal amounts of proteins (40–50 μg) per sample were resolved on 10–15% SDS-PAGE as per respective molecular weight and transferred to a 0.2 μm PVDF membrane (MDI Membrane Technologies, India). All primary antibodies were from Cell Signaling Technologies, Inc., USA. HRP-tagged secondary antibodies were from Santacruz Biotechnology, Inc., USA. Immunoblots were developed using Immobilon Forte Western HRP substrate (Merck-Millipore, USA) on an ImageQuant LAS500 chemiluminescence CCD camera (GE Healthcare, USA). Densitometry analysis was done using ImageJ 1.52 K (NIH, USA).

### Biochemical assays

The enzymatic activity of antioxidant enzymes Superoxide dismutase and Catalase was determined using the Oxiselect Superoxide Dismutase Activity assay kit (Cell Biolabs Inc. Cat# STA-340) and Catalase Assay Kit (Sigma-Aldrich, Cat# Cat100) according to the manufacturer’s instruction. GSSG/GSH levels were quantified using OxiSelect Total Glutathione Assay Kit (Cell Biolabs Inc. Cat# STA-312) following manufacturer’s instructions. The cellular ATP content was measured using the ATP determination kit (Invitrogen Ref. A22066) as per the manufacturer’s instructions.

### In-vivo experiments

Adult, 6–8 weeks old C57BL/6 mice, with an average weight of 22–26 g, were issued from the Experimental Animal Facility of INMAS, DRDO, Delhi, India. They were distributed randomly in groups of 6 per cage and kept at 22 ± 2 °C and 12–12 h/light–dark cycle and were given a standard laboratory rodent diet (Golden Feeds, Delhi, India) and water ad libitum. The power analysis to compute the sample size was done using the Power analysis tool available at the National Centre for the Replacement Refinement and Reduction of Animals in Research, London, UK (https://eda.nc3rs.org.uk). The acclimatization of animals was done one week prior to the experiments. The animals were administered bpV(HOpic) (Intraperitoneal route) 4 h before irradiation, and all animals were exposed to whole-body irradiation of 7.5 Gy using a ^60^Co source (LDI-2000; Board of Radiation and Isotope Technology, Govt. of India, Department of Atomic Energy and a dose rate of 1.723 Gy/min), except control animals. The study protocols were reviewed and approved by the Institute of Nuclear Medicine and Allied Science’s Animals Ethics Committee (IAEC), Ethical Committee Number: INM/IAEC/2018/21. All experiments were performed in accordance with relevant guidelines and regulations of the Animal Ethics Committee.

### Statistical analysis

All experiments were carried out in triplicate twice. Data were analyzed using GraphPad Prism (Version 7.0a for Mac OSX), and the experimental results were expressed as mean ± SD. Student’s t-test was performed to determine the statistical significance between the groups. Results were considered significant at *p* < 0.05. Animal survival data were analyzed using the Kaplan–Meir method followed by Mantel–Cox (log-rank test), long rank test for trend and Gehan–Breslow–Wilcoxon tests for assessment of significance differences. Results at *p* < 0.05 were considered significant.

### Ethical approval

All animal experiments in the study were performed under the protocols reviewed and approved by the Institute of Nuclear Medicine and Allied Science’s Animals Ethics Committee (IAEC), Ethical Committee Number: INM/IAEC/2018/21. Animal experiments were conducted in compliance with the principles stated in the Guide for the Care and Use of Laboratory Animals, National Research Council (US), Institute for Laboratory Animal Research, 1996.

## Results

### PTEN inhibitor, bpV(HOpic)confers resistance against IR

**Figure 1:**
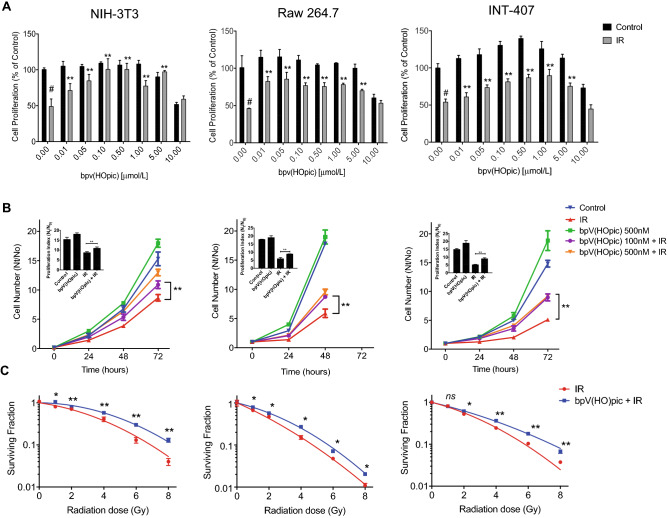
bpV(HOpic) confers radioresistance in cells. (**A**) Effect of bpV(HOpic) on radiation induced cytotoxicity was assessed by SRB assay in NIH-3T3, Raw 264.7 and INT407 cells 48 h post IR exposure. Data presented as cell proliferation % of control. (**B**) Effect of pretreatment of 100 nmol/L and 500 nmol/L concentrations of bpV(HOpic) on the proliferation index (PI)(N_t_/N_0_) of irradiated NIH-3T3 (4 Gy), Raw 264.7 (2 Gy) and INT 407 cells (6 Gy). Inset bar charts represents PI of the ultimate timepoint of each cell line. (**C**) Clonogenic survival assay of 100 nmol/L bpV(HOpic) pretreated cells exposed to different doses of irradiation. Surviving fractions of un-irradiated and irradiated samples was calculated by considering the plating efficiency of un-irradiated control as 1. Error bars are mean ± SD, with n = 4. **#** = IR versus unirradiated control (*p* < 0.01) *p < 0.05; ** *p* < 0.01; ns = non significant.

To investigate the cytoprotective potential of PTENi bpV(HOpic) against IR, we first evaluated the safe dose of PTENi in cell lines of embryonic fibroblast (NIH-3T3), hematopoietic (Raw 264.7), and gastrointestinal (GI) (INT-407) tissue origin. In humans, hematopoietic-acute radiation syndrome (H-ARS) usually manifests at 1–2 Gy, while GI-ARS at doses exceeding 6 Gy, we chose IR doses of 2 Gy for Raw 264.7 cells and 6 Gy for INT-407 cells^2, 38^. The IR dose of 4 Gy for NIH-3T3 was determined based on the existing literature on the radiation response of fibroblasts^39–41^. The radiation-induced cytotoxicity and proliferation index was assessed by SRB and growth kinetics assays respectively at indicative timepoints to evaluate the radioprotective potential of PTENi. The bpV(HOpic) was shown to be cytoprotective against IR up to 5 μmol/L in NIH-3T3, Raw 264.7, and INT-407 cell lines. The drug-induced toxicity was not apparent up to 5 μmol/L in the cell lines we tested; however, the drug alone showed toxicity at 10 μmol/L in all the three cell lines (Fig. 1A). The proliferation index of irradiated cells was compared with cells treated with two best cytoprotective concentrations of bpV(HOpic) (100 nmol/L and 500 nmol/L) one hour before irradiation. A time-dependant increase in proliferation index was observed in all three cell lines we tested when treated with bpV(HOpic), 1 h before irradiation as compared to their respective controls (Fig. 1B). In clonogenic assays also, increased clonogenicity was evident in all three irradiated cells treated with bpV(HOpic) in similar experimental conditions (Fig. 1C). Taken together, these results demonstrate that bpV(HOpic) induces radioresistance in cells.

### PTEN inhibition protects from IR-induced cell death

The effect of bpV(HOpic) on radiation-induced apoptosis was assessed using AO/EtBr staining, Annexin V/PI staining, Caspase activity, and western blot analysis of key apoptotic proteins. For this and subsequent mechanistic studies, we selected the NIH-3T3 cell model as bpV(HOpic) treatment showed good cellular protection against radiation injury in this cell line.

**Figure 2:**
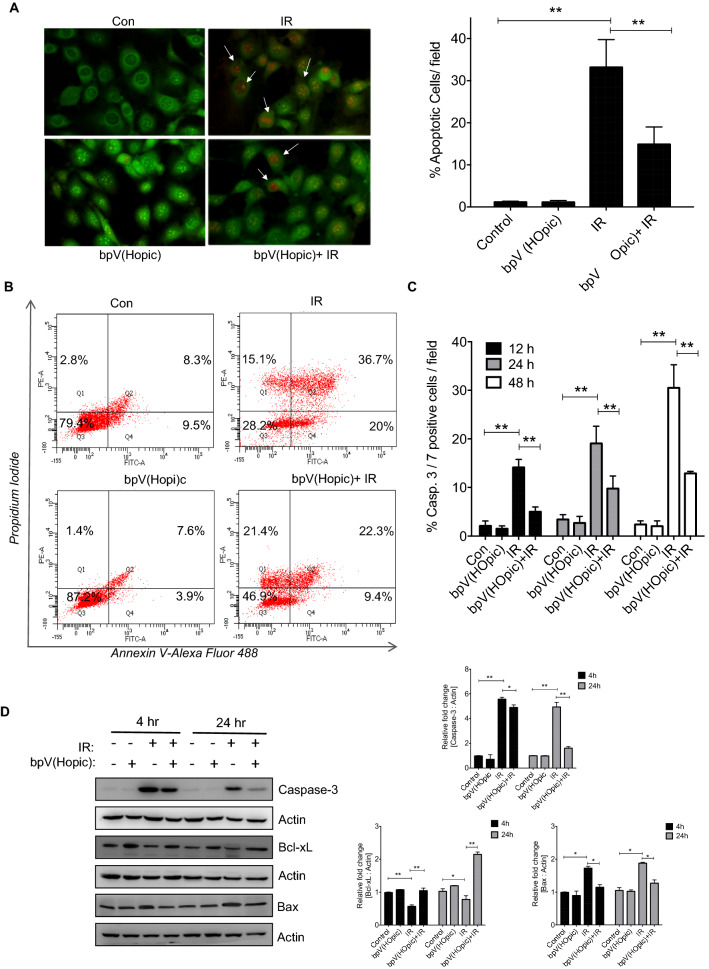
bpV(HOpic) pretreatment ameliorates radiation induced cell death. (**A**) Representative photomicrograph of AO/EtBr stained NIH-3T3 pretreated with 100 nmol/L of bpV(HOpic), 48 h post irradiation. Barcharts represents % of total EtBr positive cells per field. (**B**) Representative flow cytometry scatterplots depicting Annexin-V (Alexa Fluor 488)/Propidium Iodide staining of NIH-3T3 cells after 100 nmol/L bpV(HOpic) pretreatment and irradiation (48 h post 4 Gy IR exposure). Quadrants (Q1–Q4) represents retention of different apoptotic phase events (%). The percentages of cells indicated in each quadrant as the means ± SD (n = 4) from two independent experiments. (**C**) Caspase-3/7 activity in NIH-3T3 cells pretreated with bpV(HOpic) one hour prior to 4 Gy IR exposure assessed at at indicated time intervals using CellEvent probe and % of total Caspase-3/7 positive cells per field are represented as bar charts. Error bars are mean ± SD (n = 4) from two independent experiments. *p < 0.05; **p < 0.01. (**D**) NIH-3T3 cells were pretreated with bpV(HOpic) 1 h before irradiation and whole cell lysates at indicated timepoints were subjected to immunoblotting for key pro-apototic and pro-survival proteins. Fold change in protein levels with respect untreated control is represented with densitometry plots expressed as mean ± SD. *p < 0.05; **p < 0.01.

The appearance of condensed or fragmented orange chromatin upon EtBr uptake, a characteristic of apoptotic cells that have lost their membrane integrity, was pronounced in γ-irradiated cells at 48 h post-exposure (40.23% ± 3.9 vs. 1.1% ± 0.2 in control cells; *p* < 0.01; Fig. 2A), which was diminished by the pretreatment of bpV(HOpic) (14.1% ± 3.79; *p* < 0.01; Fig. 2A). A similar trend was observed with Annexin V/PI flow cytometric analysis, where γ-irradiation lead to increased apoptosis at 48 h post-irradiation. In contrast, bpV(HOpic) conferred protection against radiation-induced apoptosis and reduced the percentage of the late apoptotic population (37% to 22%). However, the total apoptotic population (both late and early) was reduced to 32% in bpV(HOpic) treated cells as compared to 57% in radiation alone treatment (Fig. 2B). We also assessed the caspase-3 and -7 activity as well as the protein levels of cleaved caspase-3 in response to radiation. We observed that significantly low percentage (nearly 4.9% ± 1.01 at 12 h; 9.7% ± 2.6 at 24 h; 12.9% ± 0.4 at 48 h; *p* < 0.01; Fig. 2C) of bpVHOpic pretreated cells showed caspase-3 and -7activity as compared to radiation alone treated cells, in which a high number of cells showed caspase-3 and -7 activity at all of the timepoints we assayed (14.14% ± 1.64 vs. 2.1% ± 1.03 of control at 12 h; 19% ± 3.6 vs. 3.4% ± 0.99 at 24 h; 30.5% ± 4.7 vs. 2.4% ± 0.7 of control at 48 h; *p* < 0.01; Fig. 2C). A nearly threefold difference in caspase-3 cleavage was also observed between irradiated cells and cells pretreated with bpV(HOpic) before irradiation (4.5 fold ± 0.5 vs. 1.6 ± 0.13 at 24 h; *p* < 0.01; Fig. 2D). Besides, bpVHOpic pretreated cells showed a reduced level of the radiation-induced pro-apoptotic protein Bax and augmented level of pro-survival protein Bcl-xL. These results suggest that bpVHOpic pretreatment protects cells from radiation-induced apoptosis.

### bpV(HOpic) reduces IR-induced DNA damage

**Figure 3:**
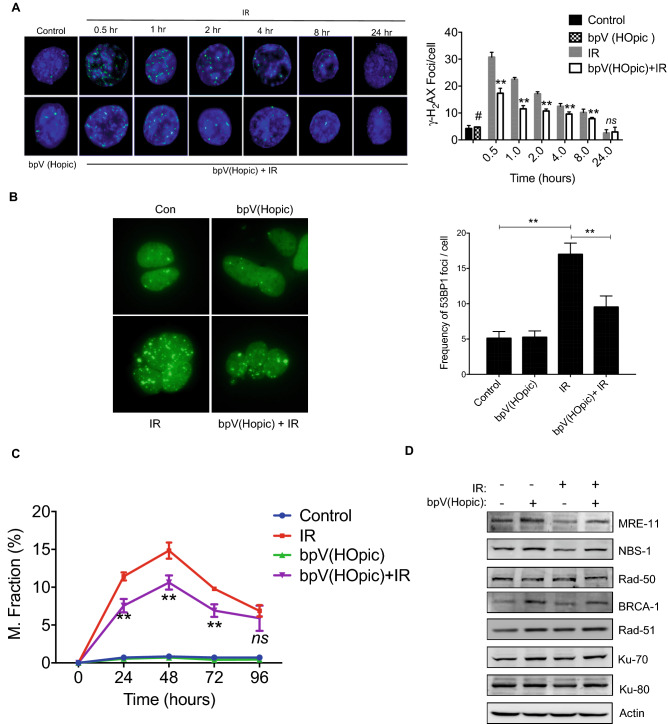
bpV(HOpic) reduces radiation induced DNA-DSBs and cytogenetic damage in NIH-3T3 cells. (**A**) Representative images of γ-H_2_AX foci formation in NIH-3T3 cells pretreated with 100 nmol/L bpV(HOpic) 1 h prior to 4 Gy IR exposure. The number of γ-H_2_AX foci per cell were scored and minimum 100 cells from each group replicate were scored. (**B**) Representative photomicrographs of 53BP-1 foci formation assay in HEK cells pretreated with 100 nmol/L bpV(HOpic) 1 h before IR exposure. Cells were stably transfected with GFP tagged 53BP-1 and were grown on coverslips. At 48 h post IR exposure, 53BP-1 foci were observed under a fluorescent microscope, and average foci per cell were scored. (**C**) IR induced cytogenetic damage (micronuclei fraction) in bpV(HOpic) (100 nmol/L) pretreated NIH-3T3 cells. (**D**) NIH-3T3 cells were pretreated with bpV(HOpic) 1 h before irradiation, and whole-cell lysates at 1-h post IR exposure were subjected to immunoblotting for indicated proteins. Error bars are mean ± SD (n = 4) from two independent experiments. # = IR vs unirradiated control (*p* < 0.01); *p < 0.05; **p < 0.01.

Besides apoptosis, radiation-induced DNA damage mediated mitotic catastrophe is another cause of death in radiation exposed cells. DSBs are the most lethal form of DNA damage; failure to resolve DSBs leads to cell death. The IR-induced DSBs are the main contributing factors for the loss of clonogenicity and increased cell death observed in cells exposed to IR^33^. Hence, the effect of PTEN inhibition on IR-induced DNA damage was assessed. The histone H2AX that undergoes phosphorylation (γH2AX) at the sites of DNA DSBs serves as a hallmark for DSBs detection. A time-dependent change in γH2AX foci, indicative of residual DNA-DSBs, was observed in NIH-3T3 cells after irradiation, detectable as early as 30 min post IR exposure (30.7 ± 1.8 vs. 4.5 ± 0.7 foci/cell in control; *p* < 0.01; Fig. 3A). However, bpV(HOpic) pretreatment efficiently reduced the number of detectable residual γH2AX foci per cell after IR exposure at 30 min (17.3 ± 1.9 foci/cell vs. 30.7 ± 1.8 in the irradiated group; *p* < 0.01; Fig. 3A). Further, the number of foci was found to be significantly lower in bpV(HOpic) pretreated cells at all the time points than radiation alone (Fig. 3A). To further strengthen the results of γH2AX foci formation assay, HEK cells stably expressing 53BP1-GFP plasmid were used as a reporter assay for IR-induced DNA double-stranded breaks. Using these reporter HEK cell line, we determined the effect of PTENi on 53-BP1 activation, a critical component of the NHEJ pathway. Cells showed increased amount of 53-BP1 foci 48-h post-irradiation (17.0 ± 1.6 vs. 5.1 ± 0.95 foci/cell in control; *p* < 0.01) that was significantly decreased by bpV(HOpic) in irradiated cells (9.6 ± 1.6 foci per cell; *p* < 0.01) (Fig. 3B). Therefore, the effect of bpV(HOpic) on DNA repair kinetics is rather a generalized observation and not specific to any species or cell line. Failure to repair DSBs leads to chromosomal aberrations that are manifested as micronuclei in daughter cells after mitosis, indicating cytogenetic damage. The kinetics of micronuclei formation was followed in NIH-3T3 (until 96 h) post-irradiation. We noted a significantly reduced number of micronuclei positive cells in the bpV(HOpic) pretreated group (Fig. 3C). Moreover, the protein levels of key sensors and regulators of DNA DSBs repair pathways were also analyzed 1-h post-radiation exposure by western blot. bpV(HOpic) pre-treatment led to a significant increase in all three components of MRN complex; interestingly, only MRE-11 (1.6 fold ± 0.2 vs. IR *p* < 0.01) and NBS-1 (1.9 ± 0.3 fold vs. IR *p* < 0.01) levels were increased in irradiated NIH-3T3 cells pre-treated with bpV(HOpic). A similar trend was observed with the protein levels of key components of the HR and NHEJ DNA repair pathways (Fig. 3D and S3). These results suggest that PTEN inhibition through bpVHOpic significantly reduces radiation-induced DNA-DSBs and cytogenetic damage through enhanced DNA repair. Reduced cytogenetic damage results in enhanced clonogenicity and cell proliferation; therefore, this observation is in line with the enhanced clonogenic potential of bpV(HOpic) treated cells after radiation exposure.

### PTEN inhibitor alleviates IR-induced oxidative stress

**Figure 4:**
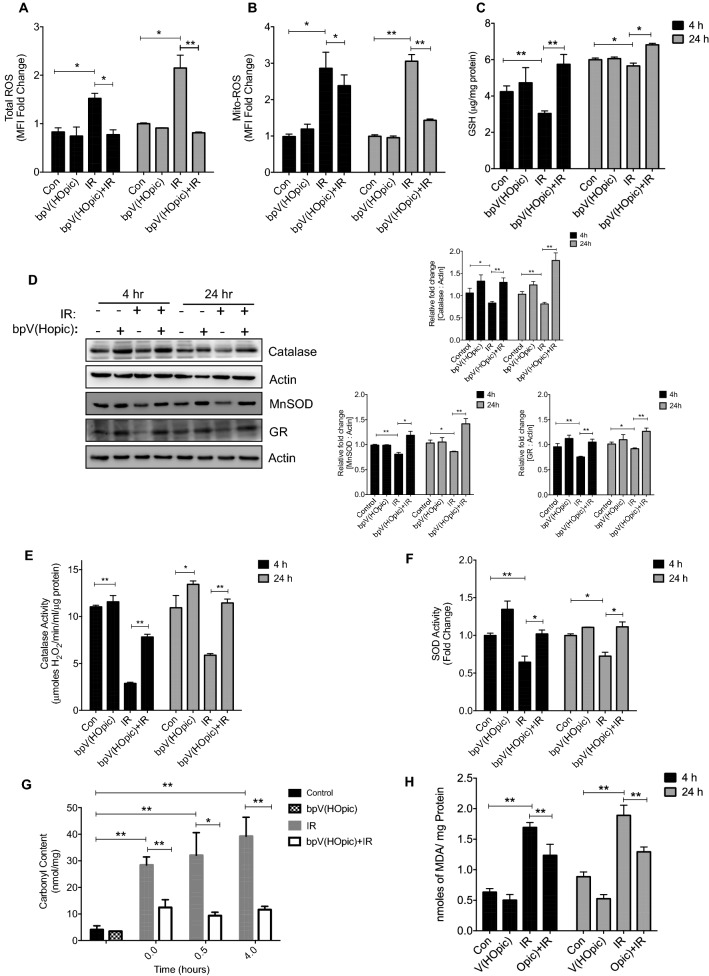
bpV(HOpic) alleviates IR-induced oxidative stress in cells by stregthening the antioxidant defense mechanism. Cell were pretreated with 100 nmol/L bpV(Hopic) 1 h before 4 Gy IR exposure (**A**) Analysis of the effect of bpV(Hopic) on total ROS production in irradiated NIH-3T3 by CM-H2DCFDA at indicated time points using flow cytometer. Bar charts represented as fold change in mean fluorescence intensity (MFI) with respective control. (**B**) Effect of bpV(Hopic) on Mitochondrial ROS production in irradiated NIH-3T3 by Mitosox Red at indicated time points using flow cytometer. Bar charts represented as fold change in mean fluorescence intensity (MFI) with respective control. (**C**) Effect of bpV(HOpic) on the levels of non-enzymatic antioxidant in irradiated NIH-3T3 cells at indicated time intervals. (**D**) After treatment, NIH-3T3 cells whole cell lysates were subjected to immunoblotting and densitometry analysis for indicated proteins of antioxidant defense mechanism. (**E**) Effect of bpV(HOpic) on the catalase activity of irradiated NIH-3T3 cells at indicated time points. (**F**) Effect of bpV(HOpic) on the SOD activity of irradiated NIH-3T3 cells at indicated time points. (**G**) Effect of bpV(HOpic) on the protein oxidation of irradiated NIH-3T3 cells assessed by nmoles of carbonyl content per mg protein at indicated time intervals. (**H**) Effect of bpV(HOpic) on the membrane lipid oxidation of irradiated NIH-3T3 cells assessed by nmoles of MDA content per mg protein at indicated time intervals. Error bars are mean ± SD (n = 4) from two independent experiments. *p < 0.05; **p < 0.01.

Since oxidative stress is the major contributing factor to the IR-induced macromolecular damage, we evaluated the role of PTENi in regulating IR-induced ROS and resulting oxidative stress. The total cellular ROS was measured at 4 and 24 h post-irradiation. Radiation exposure resulted in a significant increase in total cellular ROS at 4 h (1.5 ± 0.10 fold; *p* < 0.01; Fig. 4A), which was further augmented at 24 h (2 ± 0.27-fold; *p* < 0.01; Fig. 4A). A significant (~ twofold at 4 h and 2.6-fold at 24 h) decrease in radiation-induced total cellular ROS was observed at both early and late timepoint in cells pretreated with bpV(HOpic). Moreover, we observed nearly three-fold increased radiation-induced mitochondrial ROS in NIH-3T3 cells at 4 and 24-h post-irradiation. However, bpVHOpic pretreatment led to a marginal yet significant reduction in mitochondrial ROS at 4 h (1.2-fold). At later timepoint, a reduction in IR-induced mito-ROS levels was better (twofold vs. IR) in bpV(HOpic) pretreated cells (Fig. 4B). We also measured the level of reduced glutathione (GSH) under similar experimental conditions. PTENi alone resulted in a modest (non-significant) increase in the levels of GSH initially that were significantly reduced in irradiated cells (3.04 ± 0.14 vs. 4.24 ± 0.32 μg/mg protein in control at 4 h; *p* < 0.01; Fig. 4C). However, cells pretreated with bpV(HOpic) before irradiation showed enhanced GSH levels (5.74 ± 0.54 μg/mg protein *p* < 0.01; Fig. 4C) at 4 h and later time point also. We also examined the effect of PTENi on the protein levels of critical free radical metabolizing enzymes in irradiated NIH-3T3 cells. IR induced reduction in the levels of catalase, MnSOD, and glutathione reductase (GR) were found to be significantly replenished in co-treated cells (Fig. 4D). Consistent with this, PTENi pretreated cells also showed enhanced enzymatic activity of both catalase and total SOD enzymes, thus strengthening the radiation-induced oxidative stress modulating potential of PTENi bpV(HOpic) (Fig. 4E,F).

We further assayed the IR induced oxidative damage to protein and lipids. For protein, we followed the total cellular carbonyl content up to 4 h of post-IR exposure. We found a nearly tenfold increase in total carbonyl content in IR-exposed NIH-3T3 cells at 4 h (39.59 ± 7.16 vs. 4.11 ± 1.44 nmol/mg protein in control; *p* < 0.01; Fig. 4G). By contrast, bpV(HOpic) pretreatment leads to a 3.6-fold decrease in total carbonyl content with respect to radiation alone, 4 h post-irradiation (11.13 ± 1.21 nmole/mg protein; *p* < 0.01; Fig. 4G). At late timepoints (24 h post IR-exposure), IR elevated the levels of lipid peroxidation-end product MDA to nearly two-fold (1.89 ± 0.16 vs. 0.88 ± 0.07 nmoles/mg protein in control; *p* < 0.01), that was reduced by the bpV(HOpic) pretreatment significantly (1.29 ± 0.08 nmoles/mg protein; *p* < 0.01; Fig. 4H). These data indicated that bpV(HOpic) suppresses IR-induced oxidative stress in cells by strengthening the antioxidant defense mechanism.

### bpV(HOpic) confers radio-protection by activating AKT signaling

**Figure 5:**
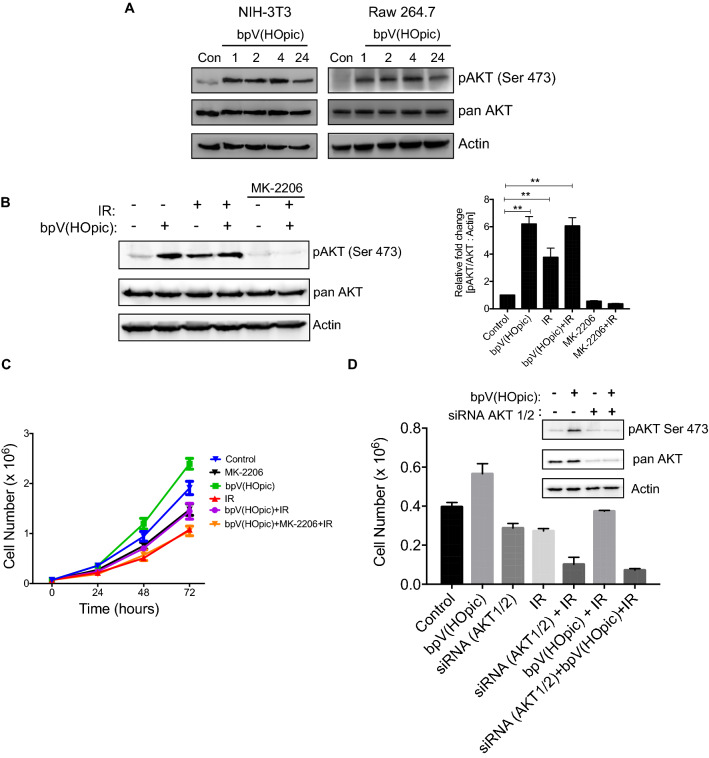
bpV(HOpic) confers radio-protection by activating AKT signaling. (**A**) NIH-3T3 and Raw 264.7 cells were treated with bpV(HOpic) for indicated timepoints and AKT activation was assessed from whole cell lysate immunoblotting for pAKT and AKT protein levels. (**B**) Effect of bpV(HOpic) pretreatment on the status of AKT signaling in irradiated NIH-3T3 cells. Cells were treated with 100 nmol/L bpV(HOpic) and/or 2.5 μM Akt inhibitor MK-2206 1 h before 4 Gy irradiation. At 1 h post-irradiation, cells were harvested, and whole-cell lysates were subjected to immunoblotting, and densitometry analysis for pAKT/AKT levels (**C**) Pharmacological inhibition of AKT signaling reverses bpV(HOpic) conferred radioresistance in NIH-3T3 cells. Cell pretreated with AKT inhibitor, MK-2206, were subjected to IR-exposure upon bpV(Hopic) treatment. Cell counts were taken at indicative timepoints for each group. (**D**) Genetic inhibition of AKT signaling reverses bpV(HOpic) conferred radioresistance in NIH-3T3 cells. Cells expressing scrambled or AKT siRNA were treated with 100 nmol/L bpV(HOpic) before 4 Gy IR exposure, and cell counts were done at indicated timepionts. Inset immunoblot represents the status of AKT signaling at the time of IR exposure. Error bars are mean ± SD (n = 4) from two independent experiments. *p < 0.05; **p < 0.01.

PTEN inhibitors are known to activate AKT signaling, and studies have shown that activated AKT signaling modulates the radiation response of malignant tissues. However, most of the studies reported the implication of AKT signaling in malignancies, and it is not clear if activated AKT signaling could modulate the radiation response of healthy tissue to a similar extent. Therefore, to assess the role of AKT, we followed the kinetics of AKT activation upon bpV(HOpic) treatment. As evident by the phospho-protein/total protein expression ratio, PTEN inhibition led to a time-dependent increase in AKT phosphorylation in NIH-3T3 as well as Raw 267.4 macrophages (Fig. 5A). Exposure to IR also results in activation of AKT as a cell survival mechanism, and the same was evident in our results where we observed a nearly fourfold fold increase in AKT phosphorylation, 1-h post-IR exposure. However, the extent of AKT activation with bpV(Hopic) pretreatment was already ~ sixfold at the time of irradiation and maintained even after 1 h post-IR exposure.

Moreover, the bpV(Hopic) and IR induced AKT activation could be reversed by pretreatment with AKT inhibitor MK-2206 (Fig. 5B). To further explore the role of AKT signaling in PTENi conferred protection against IR, we performed growth kinetics analysis after inhibiting Akt activation in irradiated NIH-3T3 cells. The pharmacological and the genetic blockage of the AKT pathway through its allosteric inhibitor MK-2206 and AKT1/2 siRNA, respectively, resulted in the reversal of the radioprotection conferred by the bpV(HOpic) in NIH-3T3 cells (Fig. 5C,D). This data indicates that bpV(HOpic) confers protection against IR by activating AKT signaling.

### bpV(HOpic) confers radioresistance by elevating glycolysis

**Figure 6:**
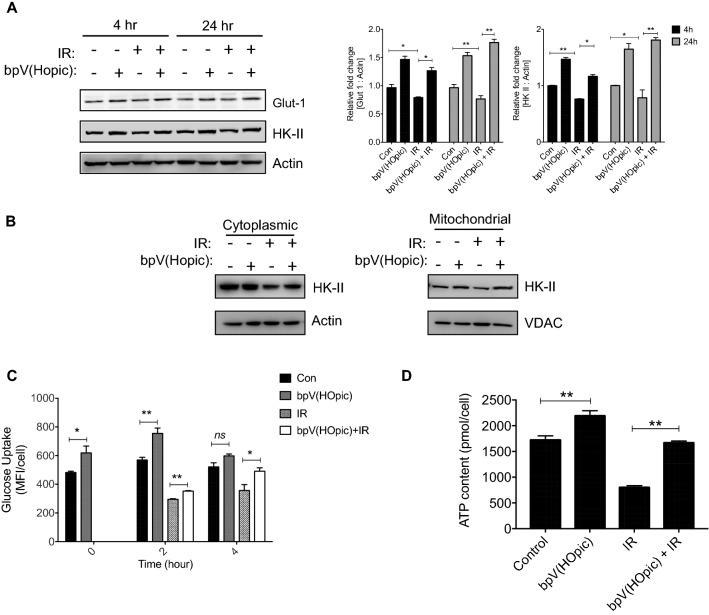
bpV(HOpic) protects cells against IR through AKT-induced enhanced glycolysis. (**A**) Effect of bpV(HOpic) on the levels of key glycolysis proteins in irradiated NIH-3T3 cells. Inset bar charts represent densitometry analysis of proteins normalized to Actin control. (**B**) Effect of bpV(HOpic) on the mitochondrial localization of Hexokinase II in NIH-3T3 cells at 24-h post IR exposure. (**C**) Glucose uptake in bpV(HOpic) pretreated 4 Gy irradiated NIH-3T3 assessed at indicated timepoints with 2-NBDG and flow cytometry. Control and bpV(HOpic) groups at 0 h represent the status of glucose uptake at the time of irradiation, represented as the mean fluorescent intensity of 2-NBDG uptake. (**D**) Effect of bpV(HOpic) pretreatment on the cellular ATP content of irradiated NIH-3T3 cells. Error bars are mean ± SD (n = 4) from two independent experiments. *p < 0.05; **p < 0.01; ns = non-significant.

AKT exerts its radio-modulating effect by increasing the expressions of key metabolic proteins, thereby increasing the glycolysis^42^ and enhanced glycolysis is also known to induce radioresistance in cells^33^. In agreement with the previous reports, increase in the levels of the key regulators of glycolysis was evident in cells treated with bpV(HOpic) where up to 1.5 fold increase in Glut 1 was observed at 4 and 24 h time intervals; and up to 1.6 fold increase in HKII was found at 4 h and 24 h as compared to their respective untreated controls; *p* < 0.01. Further, bpV(HOpic) significantly prevented the IR-induced reduction in levels of Glut 1 and HKII proteins (Glut 1 = 1.76 fold vs. 0.8 in IR at 24 h; *p* < 0.01; HKII = 1.8 fold vs. 0.7 in IR at 24 h; *p* < 0.01) (Fig. 6A). Mitochondrial translocation of HKII, the first enzyme of the glycolysis pathway, is known to increase the glycolytic flux, cell proliferation, and is critical for the regulation of mitochondria-dependant apoptosis. We estimated the levels of HKII in mitochondria by western blot analysis, where a significant decrease in mitochondrial HKII was observed in NIH-3T3 cells exposed to ionizing radiation. The reduced levels of mt-HKII were not only replenished but increased by bpV(HOpic) treatment of NIH-3T3 cells. The level of glucose uptake was followed in bpV(HOpic) treated NIH-3T3 cells post-irradiation. At the time of irradiation (one-hour post-PTENi treatment), there was a significant increase in the mean fluorescence intensity of glucose fluorescent analog 2-NBDG in PTENi treated NIH-3T3 cells (1.2-fold; *p* < 0.01). A modest yet statistically significant increase in glucose uptake was also observed at 2 h (*p* < 0.01) and 4 h (*p* < 0.05) time-intervals after irradiation in cells treated with bpV(HOpic) (Fig. 6C). Further, we also noted a net gain in total ATP content per cell in irradiated cells that received bpV(Hopic) pretreatment (1668.69 ± 31.97 vs. 801.82 ± 35.03 pmol/cell in IR; *p* < 0.01; Fig. 6D). These results indicate that bpV(HOpic) protects against the lethal effect of IR through AKT-induced enhanced glycolysis.

### PTEN inhibition confers protection against radiation in animals

**Figure 7:**
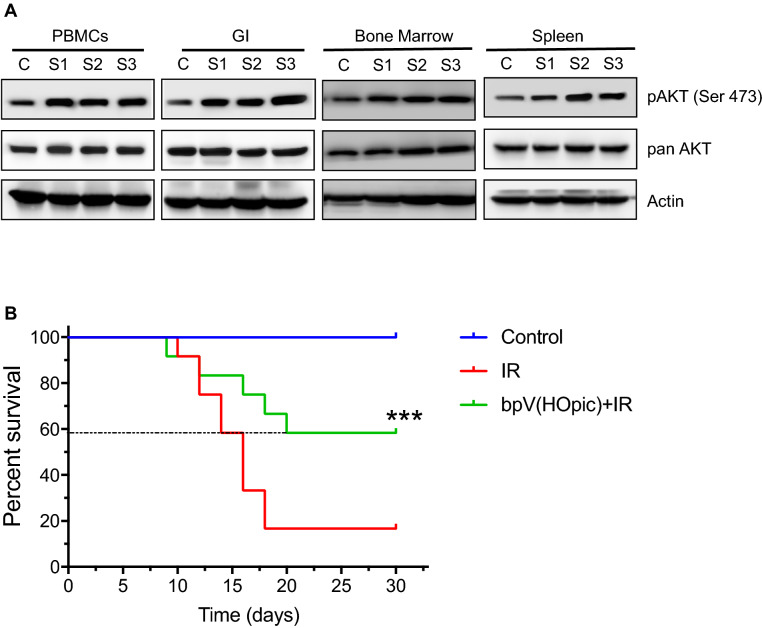
bpV(HOpic) confers protection against the lethal effect of radiation in vivo. (**A**) Effect of bpV(HOpic) on the induction of AKT signaling in vivo. Indicated tissues [Peripheral blood mononuclear cells (PBMCs); Gastro-Intestinal (GI); Bone Marrow; and Spleen] were harvested 4 h post 1 mg/Kg body weight bpV(HOpic) administration (n = 3 per group), intraperitoneally and 10% tissue homogenate was lysed and subjected to immunoblotting for pAKT and AKT levels. Lane C is a sample from control animal; Lanes S1–S3 are samples from animals 1–3. (**B**) Effect of bpV(HOpic) pretreatment (1 mg/Kg body weight; IP) on the survival of whole-body irradiated C57BL/6 mice. Statistical significance was measured using log-rank test. n = 12 per group, ***p < 0.001.

We further wanted to check whether the bpV(HOpic) induced and AKT mediated cytoprotection in vitro model can translate in vivo. For thi**s,** we administered the bpV(HOpic) (1 mg/kg body weight) intraperitoneally in C57BL/6 mice, and induction of AKT signaling in animals treated with bpV(HOpic) was investigated by immunoblotting. A significant induction of AKT signaling was evident from pAKT/AKT levels in gastrointestinal and hematopoietic tissues of bpV(HOpic) treated mice (Fig. 7A). After ensuring the bpV(HOpic) induced AKT activation in radiosensitive tissues in animals, we conducted an animal survival experiment following whole-body radiation exposure. We observed ~ 16% survival in mice exposed to a dose of 7.5 Gy whole-body irradiation, whereas bpV(HOpic) pretreatment provided survival advantage in radiation-exposed animals that was found to be significant in log-rank tests (58%; *p* < 0.001; n = 12 per group), however, the result was also found very close to significance in trend analysis by same method (*p* = 0.06) (Fig. 7B). Taken together, these findings demonstrated an in-vitro and in-vivo radioprotective potential of PTENi, bpV(HOpic).

## Discussion

Exposure to IR causes cell macromolecular damages, thereby triggering a number of cellular responses and signal transduction pathways^1, 43^. Several signaling modifier molecules reducing the extent of IR-induced macromolecular damage are currently envisaged in radioprotection. PI3K–AKT axis is a vital signaling pathway that regulates DNA repair, cell cycle checkpoints, apoptosis, and senescence to determine the fate of cells following exposure to IR^13, 16, 17, 44^. The activity of AKT is negatively regulated primarily via PTEN phosphatases by reversing the effects of PI3K action (converting PIP3 back to PIP2, through dephosphorylation of PIP3)^20, 21^. Therefore, the critical balance between PI3K and PTEN activities has a significant influence on AKT signaling and cell survival following stress exposures. Several PTEN inhibitors have been employed in oxidative stress-induced conditions where they are shown to protect the tissue from oxidative damage^22–25^. Based on this, we hypothesized that inhibition of PTEN would facilitate the DNA repair and ROS clearance, thereby protecting the cells from IR-induced cytotoxicity.

The hematopoietic and gastrointestinal systems are the most sensitive organ system to IR-induced cellular damage where exposure to doses 2–5 Gy leads to the manifestation of H-ARS and doses exceeding 5 Gy results in GI-ARS^2, 38^. Hence, oxidative damage to these organ systems is of concern for the efficacy of an effective radioprotector. Inhibition of PTEN by bpV(HOpic) one hour before IR-exposure shown to protect the cells of hematopoietic, intestinal, and fibroblast tissue origin against the radiation-induced cytotoxicity (Fig. 1A). Upon IR-exposure, the loss of clonogenicity occurs as cells are unable to divide and produce progenies owing to their lost ability to synthesize proteins and DNA; such cells are considered dead^33^. Compared with untreated cells, a gain in cell proliferation and clonogenic cell survival was evident in all three cell lines pretreated with bpV(HOpic) before IR exposure, indicating that PTENi treated cell lines showed significantly increased radioresistance (Fig. 1B,C).

IR induced macromolecular damage activates numerous cell death pathways. Where apoptosis is the majorly observed mechanism of cell death, executed through a series of molecular events. The well-characterized hallmarks of radiation-induced apoptosis^45, 46^, like pyknosis (irreversible chromatin condensation) and loss of plasma membrane asymmetry and integrity, were markedly evident in our results that were abrogated by bpV(HOpic) treatment (Fig. 2A). Our data also indicated a notable reduction in total apoptotic cells (early and late) in bpV(HOpic) pretreated cells when compared to radiation alone treated cells (Fig. 2B). Activation of caspases through their proteolytic cleavage is critical for the execution of apoptosis. This correlated with our results of a high amount of effector caspases-3 and -7 activity and cleaved caspase 3 protein levels in cells exposed to IR. The bpV(HOpic) treatment shown to reduce the caspases activity as well as protein levels of activated caspases (Fig. 2C,D). A significant reduction of pro-apoptotic proteins (Bax) and an increase in anti-apoptotic protein (Bcl-xL) levels in drug-treated groups indicated that bpV(HOpic) plays a protective role by reducing the radiation-induced apoptosis (Fig. 2D).

IR induces double-strand DNA breaks, which are potentially lethal and leads to cell death. Radiosensitive cells also lack efficient DNA repair capacity, those cells which escape apoptosis even after accumulating radiation-induced DNA damage die through mitotic catastrophe linked cell death^33, 47, 48^. Therefore, DNA repair plays a crucial role in regulating radiation-induced cell death. The observed reduction in cell death could be due to the better and faster DNA repair process in groups pretreated with PTEN inhibitors. Several lines of evidence exist where the direct and indirect role of the PI3K–AKT pathway has been shown to have implication in DNA repair processes^7, 18, 49–51^. The activation of the PI3K–AKT pathway due to the inactivation of PTEN has been correlated with radioresistance^52, 53^. Our results corroborated with these studies where we observed a faster and better clearance of IR induced residual DNA lesions (Fig. 3A,B). The components of DNA repair pathways are shown to be under the regulation of PI3K–AKT axis^54–56^. The bpV(HOpic) pretreatment led to an increased induction and activation in the key components of the DNA DSB repair pathways (Fig. 3D and S3), which could be one possible mechanism for the faster and better clearance of DNA lesions in irradiated cells. Aberrant mitosis generates abnormal chromosome segregation, which on cell division leads to the formation of nuclear anomalies like micronuclei^47^. Time kinetics of micronuclei expression in irradiated cells clearly revealed that bpV(HOpic) pretreatment significantly reduced the number of micronuclei (Fig. 3C). Further, increased proliferation index, clonogenicity, and reduced cell death observed with bpV(HOpic) pretreatment (Figs. 1, 2) could be attributed to this.

The detrimental biological effects of IR are primarily orchestrated through oxidative stress, where exposure of cellular water to IR generates potent oxidants viz. reactive oxygen species (ROS) and reactive nitrogen species (RNS). Failure of cellular antioxidant defense mechanisms to metabolize these oxidants results in the oxidation of biomolecules like lipids, proteins, and DNA^1^. Exposure to IR resulted in a significant amount of oxidative stress that was evident in the form of increased total and mitochondrial ROS, reduced glutathione levels, key antioxidant enzymes protein levels, and their activity; oxidized total cellular protein and lipid content (Fig. 4A–H). The accumulating oxidative stress, due to increased amounts of free radicals generated by IR exposure, is the major contributing factor for the macromolecular damage, culminating in cell death/apoptosis. The increased levels of oxidized biomolecules viz. lipids and proteins observed in IR exposed cells could be due to the free radical-mediated damage to the cell membranes and proteins, that further contributes to reduced enzymatic antioxidant defense systems (Fig. 4). These observations have direct implications in the high levels of residual DNA damage and cell death in IR exposed cells. Exposure to IR also results in the perturbations of the electron transport chain leading to mitochondrial ROS overproduction, further contributing to mitochondrial ROS-mediated oxidative stress. Pretreatment of bpV(HOpic) reduced the IR-induced oxidative stress and also replenished the enzymatic (Catalase and SOD) and non-enzymatic (GSH) antioxidant defense systems in cells. Since oxidative stress is a major driving factor of IR-induced cytotoxicity, our observations suggest that the bpV(HOpic) ability to reduce IR-induced oxidative stress may be the primarily responsible factor for the reduction in the DNA damage and subsequently, cell death.

Several lines of evidence exist where the PI3K–AKT axis has been shown to regulate cellular responses to IR, and transient activation of AKT signaling was a major driving factor to our hypothesis in this study to confer protection against the deleterious effects of IR. To explore the role of AKT signaling in bpV(HOpic) conferred radioprotection against IR, we examined the induction of AKT signaling post-bpV(HOpic) administration in cells and animals. Significant induction of AKT signaling could be seen as early as one-hour post bpV(HOpic) administration, both in-vitro and in-vivo. IR is also known to induce AKT signaling, and radiation-induced AKT phosphorylation (Fig. 5B) can be seen as a cellular stress response. However, by the time AKT activation takes place after radiation exposure, the pro-death pathway may dominate and overcome the AKT induced pro-survival pathways like antioxidant defense mechanisms and DNA repair pathways. On the other hand, preactivation of AKT signaling in bpV(HOpic) pretreated cells can prepare the cells to cope up with the radiation-induced oxidative and DNA damaging stress. Further inhibition of AKT phosphorylation using MK2206 reverts the bpV(HOpic) induced radioresistance in NIH-3T3 cells, suggesting the role of AKT in PTENi induced radioresistance (Fig. 5).

Cells undergoing DNA repair process require a continuous supply of energy as DNA repair is indeed a completely ATP dependant process. In our previous study^33^, we have shown that cells with stimulated glycolysis have faster kinetics of IR-induced DNA lesions clearance. The potential role of the PI3K/AKT signaling pathway in stimulating glycolysis is well documented, where AKT regulates multiple steps in glycolysis that include inducing glucose transporters (GLUT) gene expression and enhancing hexokinase activity by translocating it to mitochondrial outer membrane^17, 57–60^. AKT induced hexokinase activity has a key role in regulating glucose uptake by converting glucose to glucose-6-phosphate^61^. In agreement with this, our results showed significant induction in the protein levels of Glut-1 and HKII, as well as glycolysis with the bpV(HOpic) treatment, which results in a net increase in cellular ATP (Fig. 6A–D). In addition to that, HKII is also known to play a role in regulating the apoptosis by binding to the mitochondria and inhibiting the Bax-induced cytochrome c release^62^. In our results also a significant increase in mitochondrial bound HKII was observed (Fig. 6B), which might be responsible for reduced IR-induced cell death in bpV(HOpic) treated cells. The faster DNA repair kinetics and diminished cell death observed in bpV(HOpic) pretreated cells could be attributed to the enhanced glycolysis (Fig. 6C), resulting in a net reduction in mitotic catastrophe/apoptosis and enhanced clonogenicity as evident in our study.

With regard to this, we demonstrated here that pharmacological inhibition of PTEN protects against the lethal effect of radiation through AKT activation. Our study shows that bpV(HOpic) confers radioprotection by enhanced ROS clearance through better antioxidant signaling and faster DNA repair, thereby reducing the residual DNA damage and mitotic death. Furthermore, a single dose pretreatment of PTENi resulted in a declined cell death in an animal cell model and conferred a survival advantage to animals. However, inducing oncogene AKT by inhibition of PTEN could be the limitation for using this molecule as a radio-protector. Still, transient inhibition of PTEN has been investigated previously, where up to F2 progenies were found to be free from any tumors or other forms of chronic illness^32^. In line with this, our findings gained further support from the animal survival studies where bpV(HOpic) showed a very good trend in radio-protection, conferring 58% survival against IR. However, further investigation on large sample size, dose, route, and time of administration before IR exposure needs further exploration to derive any conclusions and further enhance the radio-protective effect of bpV(HOpic). Taken together, these findings clearly suggested that PTEN inhibition has the potential of alleviating IR induced cell cytotoxicity in an AKT dependent manner. To our knowledge, this is the first report of PTENi usage in counteracting the radiation-induced cellular damage. Further understanding of the mechanism of bpV(HOpic) conferred radioprotection in vivo will pave the way for utilizing PTEN inhibition as a possible target for the development of radiation countermeasure drugs.

## Supplementary Information

Supplementary Figures
