# Supplementary material for: PTEN inhibitor bpV(HOpic) confers protection against ionizing radiation
Source: Sci Rep. 2021 Jan 18;11:1720. doi: 10.1038/s41598-020-80754-8 (PMC7814022; doi:10.1038/s41598-020-80754-8)
Supplement: Supplementary file 1 — Supplementary Figures [file 41598_2020_80754_MOESM1_ESM.pdf]

Supplementary material- PTEN inhibitor bpV(HOpic) confers protection against ionizing radiation

Ankit Chauhan[1], Dhananjay Kumar Sah[1], Neeraj Kumari[1], Namita Kalra[1], Ravi Soni[1] and Anant Narayan Bhatt[1]*

[1]Institute of Nuclear Medicine & Allied Sciences, Delhi, India.

***Corresponding author:**
Dr. Anant Narayan Bhatt
E-mail: anbhatt@yahoo.com; anant@inmas.drdo.in
Institute of Nuclear Medicine and Allied Sciences,
Brig. S. K. Mazumdar Road, Timarpur,
Delhi-110 054, India.

Figure S1

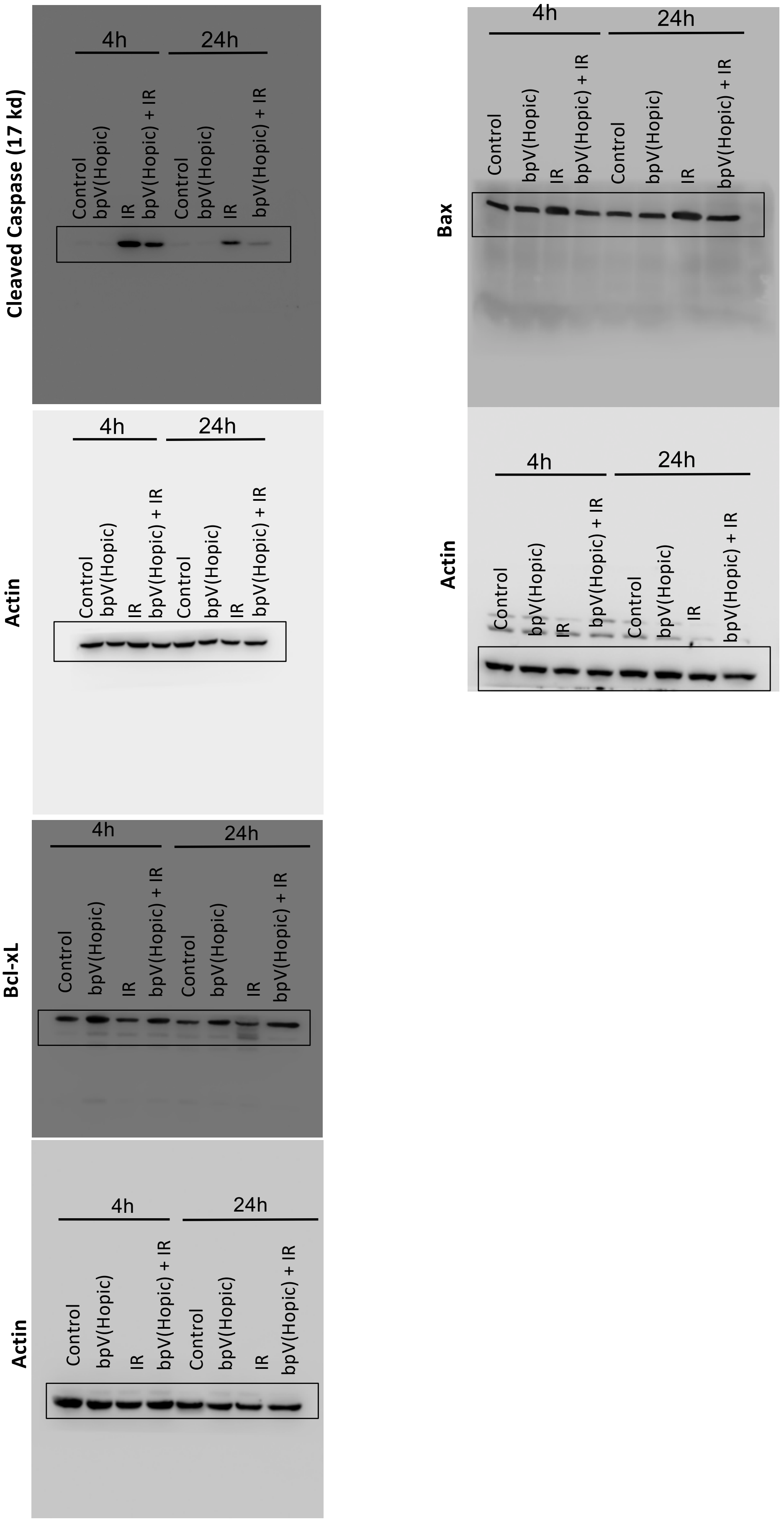

Supplementary Figure S1: Full length immunoblots of proteins and their respective loading controls represented in Figure 2D.

Figure S2

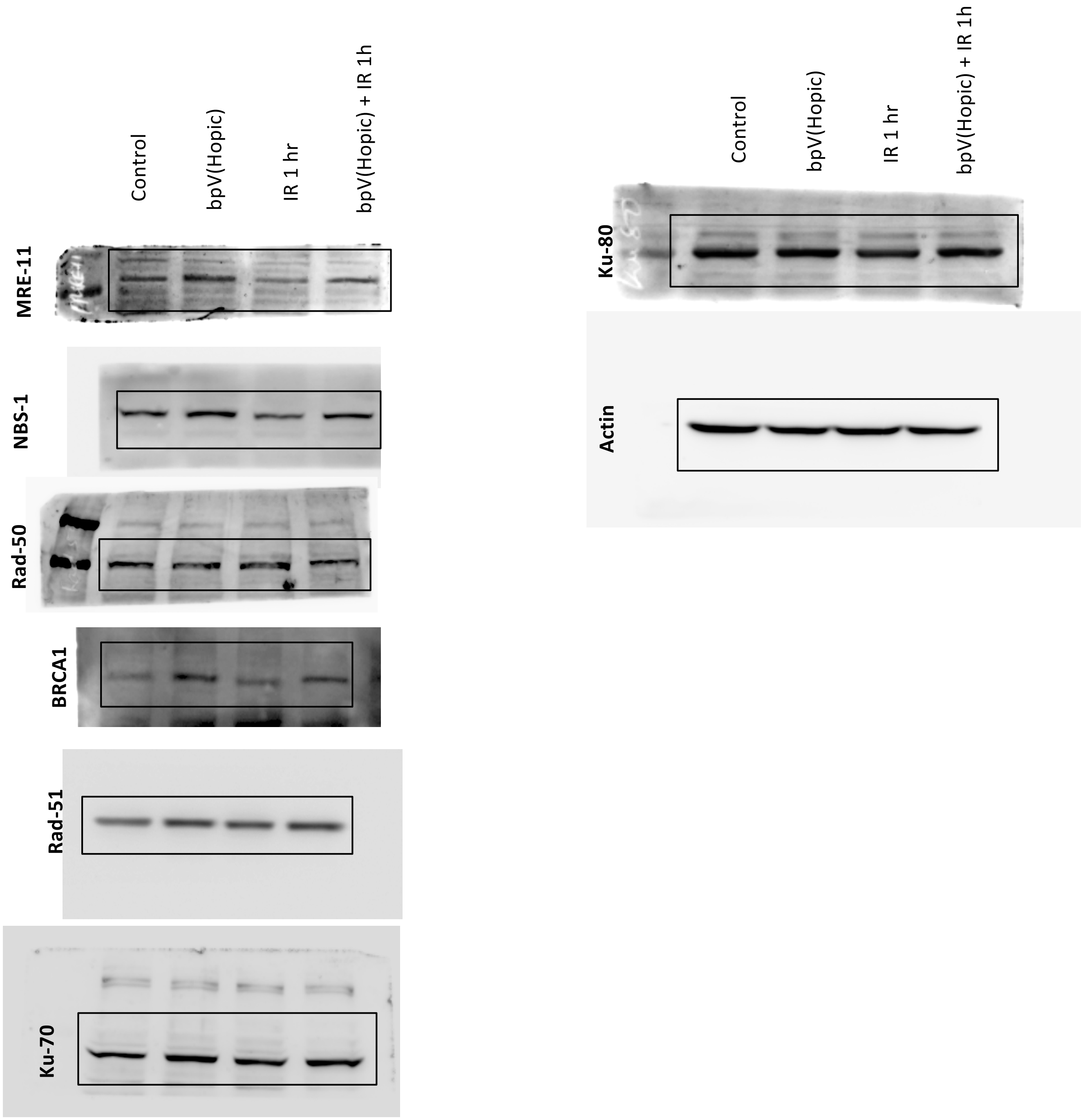

Supplementary Figure S2: Full length immunoblots of proteins and their respective loading controls represented in Figure 3D.

Figure S3

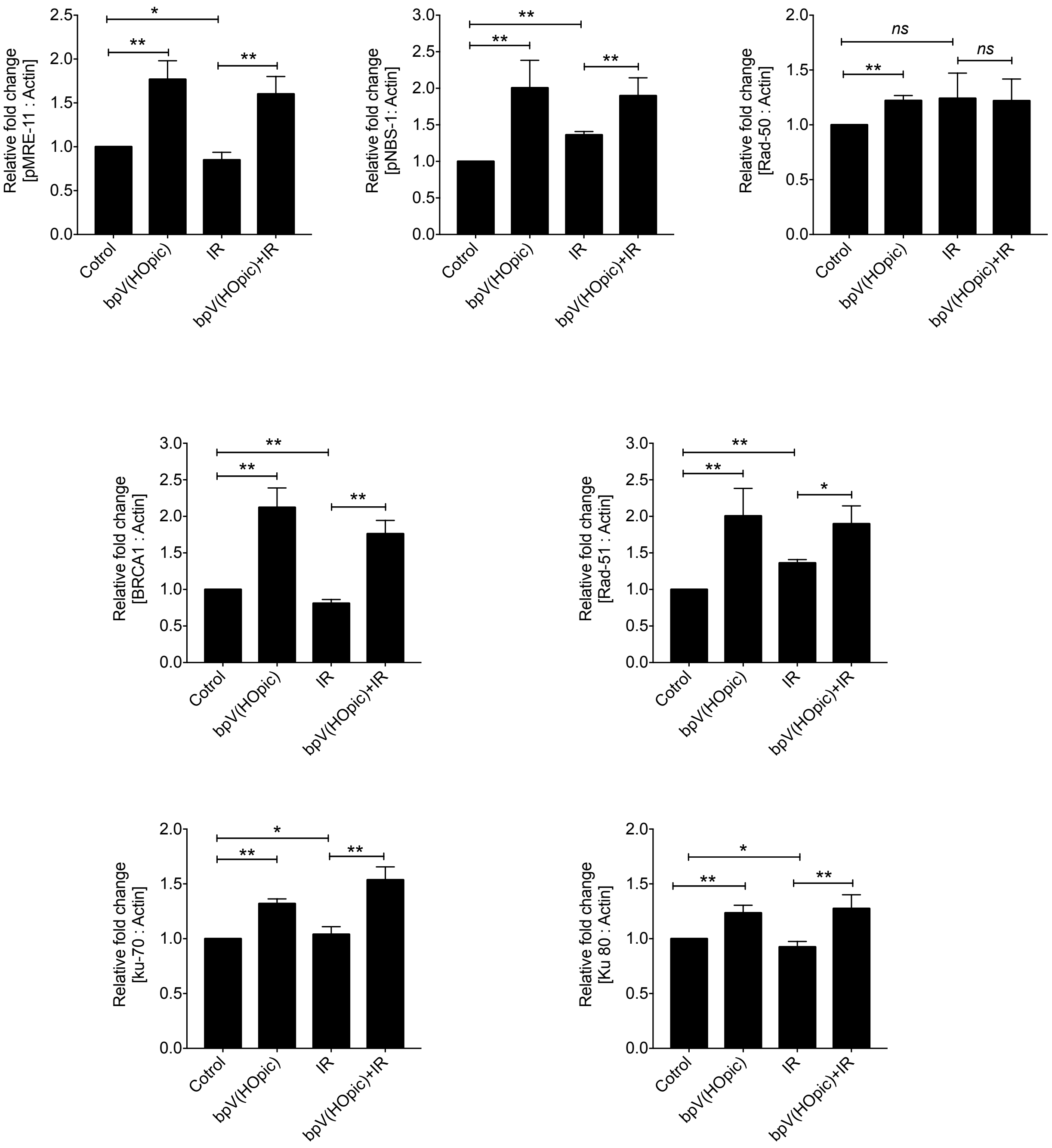

Supplementary Figure S3: Densitometry plot of DNA repair proteins normalized their respective loading controls represented in Figure 3D.

Figure S4

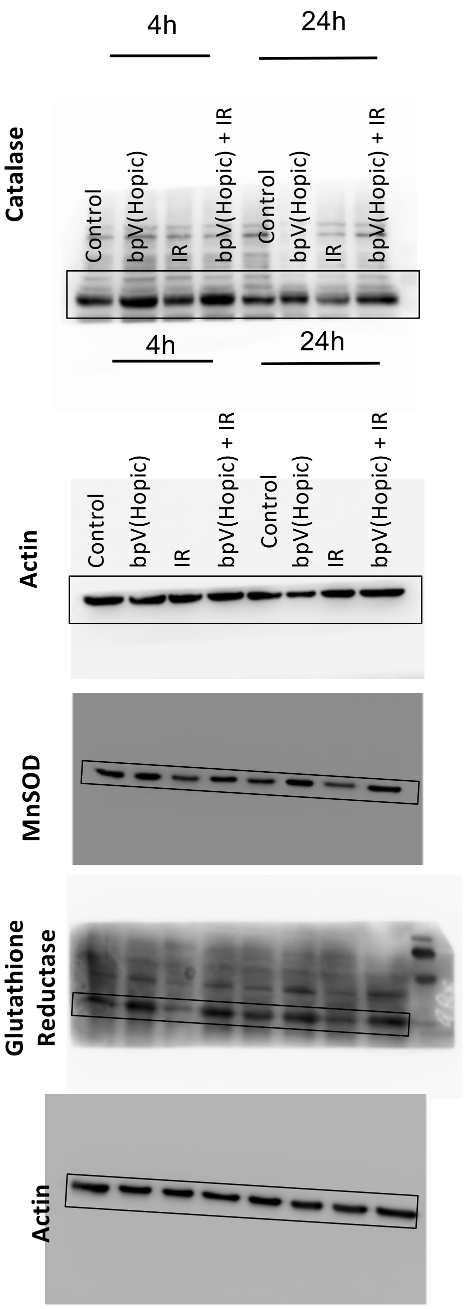

Supplementary Figure S4: Full length immunoblots of proteins and their respective loading controls represented in Figure 4D.

Figure S5

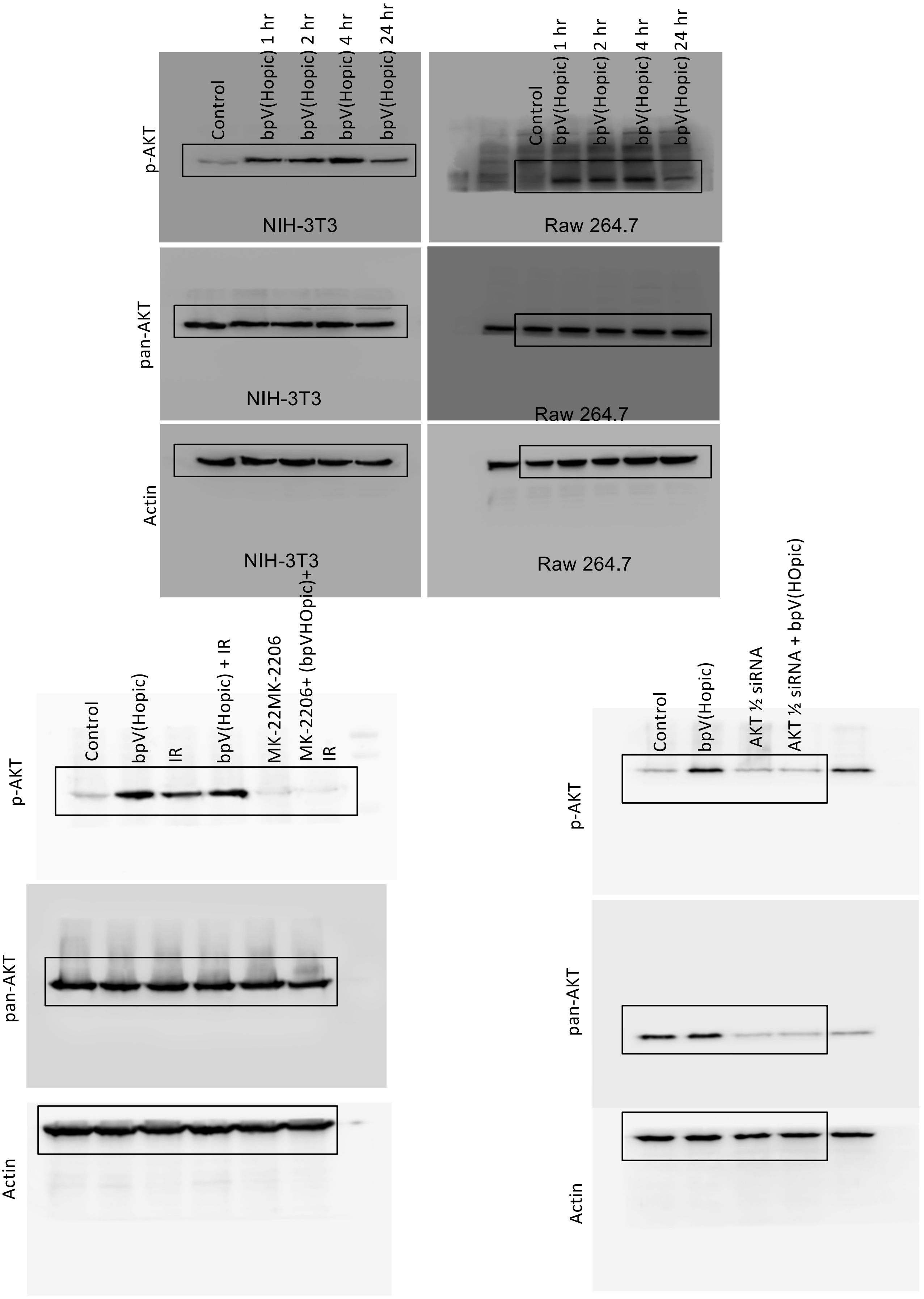

Supplementary Figure S5: Full length immunoblots of proteins and their respective loading controls represented in Figure 5.

Figure S6

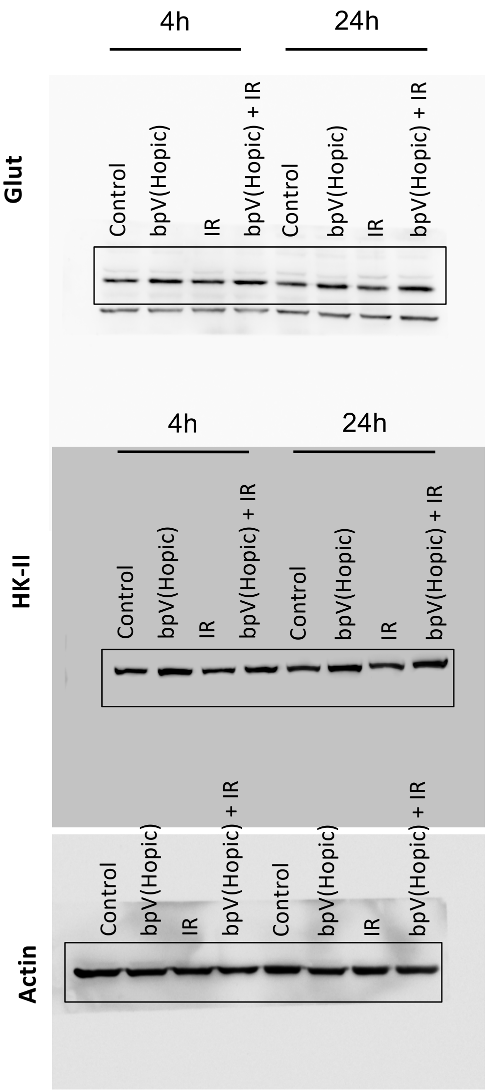

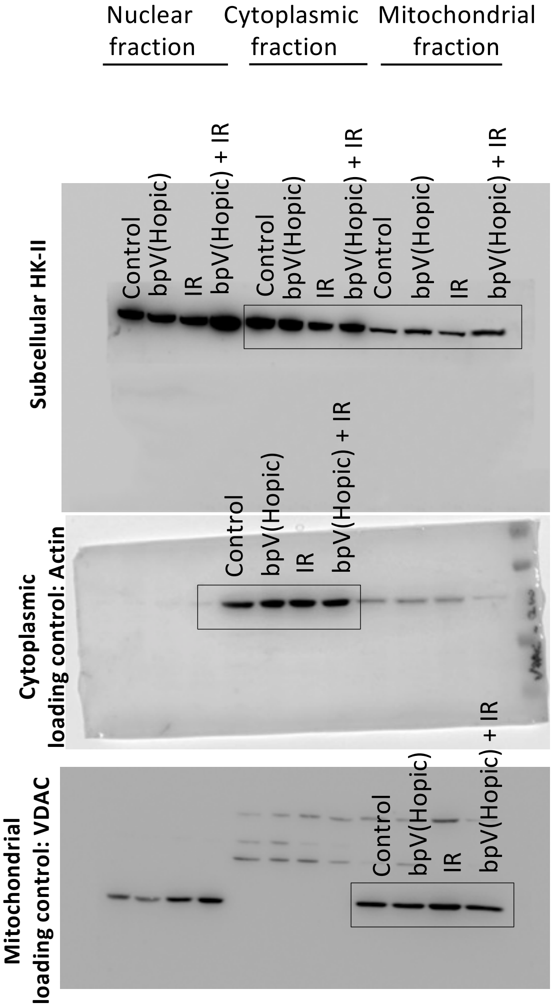

Supplementary Figure S6: Full length immunoblots of proteins and their respective loading controls represented in Figure 6.

Figure S7

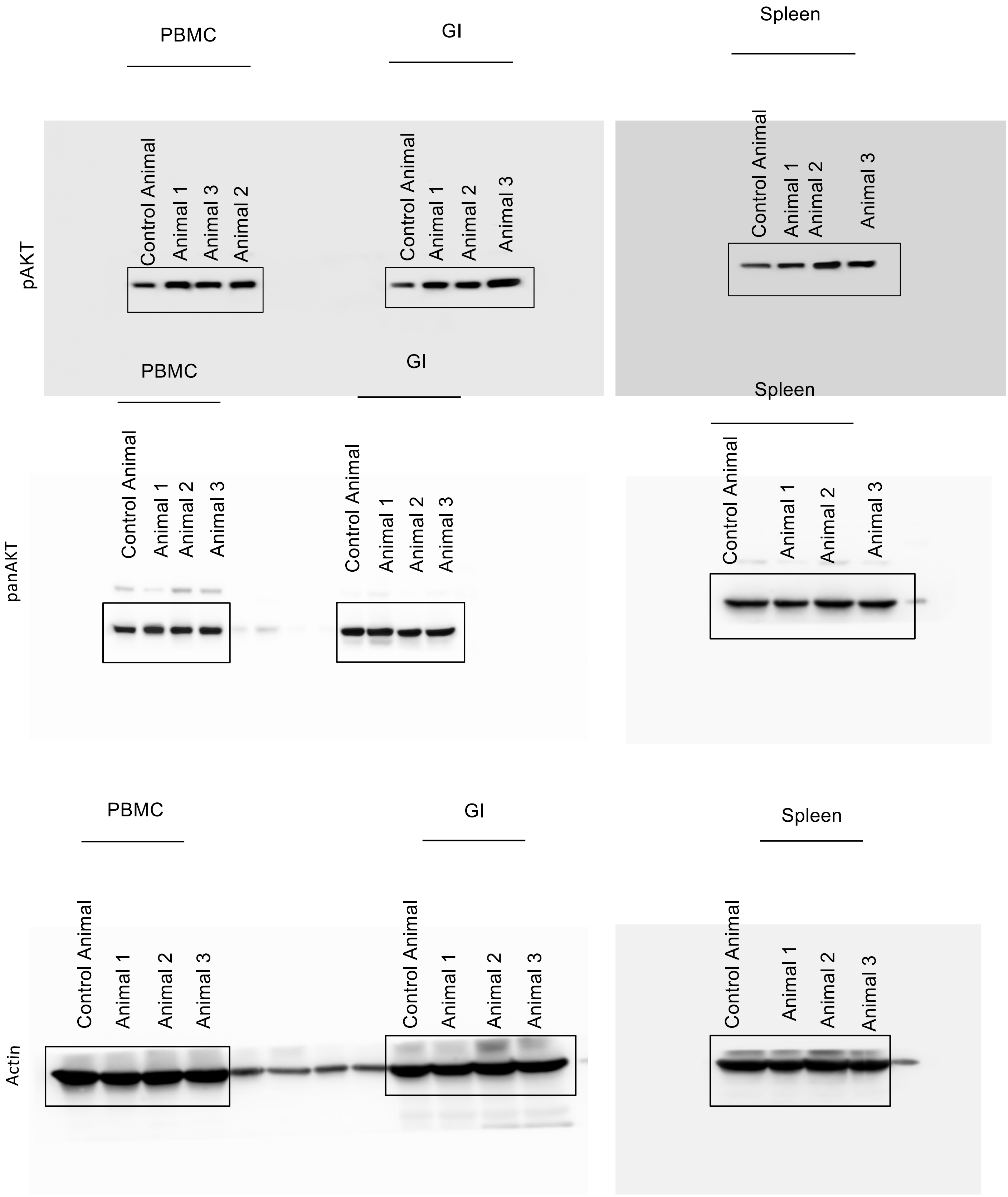
PBMC
GI
Spleen
pAKT
Control Animal
Animal 1
Animal 3
Animal 2
Control Animal
Animal 1
Animal 2
Animal 3
Control Animal
Animal 1
Animal 2
Animal 3
PBMC
GI
Spleen
panAKT
Control Animal
Animal 1
Animal 2
Animal 3
Control Animal
Animal 1
Animal 2
Animal 3
Control Animal
Animal 1
Animal 2
Animal 3
PBMC
GI
Spleen
Actin
Control Animal
Animal 1
Animal 2
Animal 3
Control Animal
Animal 1
Animal 2
Animal 3
Control Animal
Animal 1
Animal 2
Animal 3

Figure S7 continued

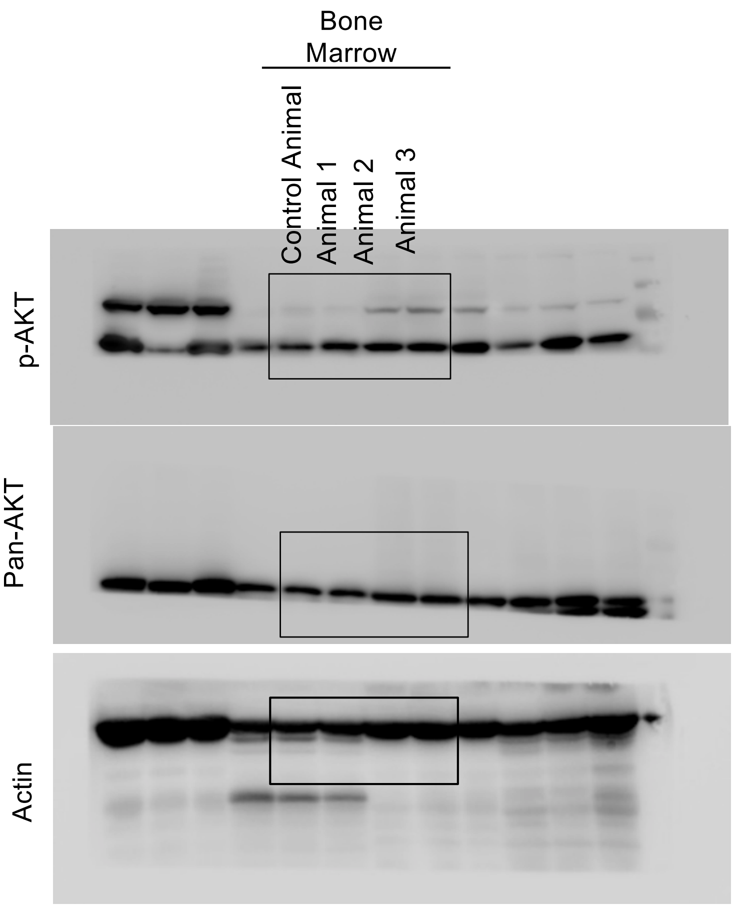

Supplementary Figure S7: Full length immunoblots of proteins and their respective loading controls represented in Figure 7.
